# The M-phase specific hyperphosphorylation of Staufen2 involved the cyclin-dependent kinase CDK1

**DOI:** 10.1186/s12860-017-0142-z

**Published:** 2017-07-14

**Authors:** Rémy Beaujois, Elizabeth Ottoni, Xin Zhang, Christina Gagnon, Sami HSine, Stéphanie Mollet, Wildriss Viranaicken, Luc DesGroseillers

**Affiliations:** 1 Present address: UMR PIMIT, Processus Infectieux en Milieu Insulaire Tropical, Université de la Réunion, 97490 Sainte Clotilde, La Réunion, France; 2 Département de biochimie et médecine moléculaire, Faculté de médecine, Université de Montréal, 2900 Edouard Montpetit, Montréal, QC H3T 1J4 Canada

**Keywords:** Staufen2, Cell cycle, Mitosis, Cyclin-dependent kinase, Phosphorylation, RNA-binding protein

## Abstract

**Background:**

Staufen2 (STAU2) is an RNA-binding protein involved in the post-transcriptional regulation of gene expression. This protein was shown to be required for organ formation and cell differentiation. Although STAU2 functions have been reported in neuronal cells, its role in dividing cells remains deeply uncharacterized. Especially, its regulation during the cell cycle is completely unknown.

**Results:**

In this study, we showed that STAU2 isoforms display a mitosis-specific slow migration pattern on SDS-gels in all tested transformed and untransformed cell lines. Deeper analyses in hTert-RPE1 and HeLa cells further indicated that the slow migration pattern of STAU2 isoforms is due to phosphorylation. Time course studies showed that STAU2 phosphorylation occurs before prometaphase and terminates as cells exit mitosis. Interestingly, STAU2 isoforms were phosphorylated on several amino acid residues in the C-terminal half via the cyclin-dependent kinase 1 (Cdk1), an enzyme known to play crucial roles during mitosis. Introduction of phospho-mimetic or phospho-null mutations in STAU2 did not impair its RNA-binding capacity, its stability, its interaction with protein co-factors or its sub-cellular localization, suggesting that STAU2 phosphorylation in mitosis does not regulate these functions. Similarly, STAU2 phosphorylation is not likely to be crucial for cell cycle progression since expression of phosphorylation mutants in hTert-RPE1 cells did not impair cell proliferation.

**Conclusions:**

Altogether, these results indicate that STAU2 isoforms are phosphorylated during mitosis and that the phosphorylation process involves Cdk1. The meaning of this post-translational modification is still elusive.

**Electronic supplementary material:**

The online version of this article (doi:10.1186/s12860-017-0142-z) contains supplementary material, which is available to authorized users.

## Background

Cell cycle can be defined as a succession of events that allow cell to replicate DNA and segregate chromosomes into two daughter cells. Proper proceeding through each step of the cell cycle depends on the expression and activity of many critical proteins such as proto-oncogenes, tumour suppressors and other regulators [1]. When mis-regulated by mutations, these effectors cause cell proliferative disorders, genomic instability and cell injuries leading to tumour emergences [2]. Among the crucial regulators of the cell cycle are the evolutionarily conserved cyclin-dependant kinases (Cdk). Cell-cycle dependent activation of members of this important family of serine/threonine kinases is mediated by their association with sequentially expressed cyclin partners [3–8]. The control of the checkpoint pathways is granted by the balance between protein synthesis and degradation and by post-translational modifications of its effectors.

The most fundamental and dominant mechanism of post-translational modifications in eukaryotes involves site-specific protein phosphorylation. The reversible transfer of a γ-phosphate to threonine, serine or tyrosine residues is mediated by a super-family of protein kinases that causes conformational changes to the proteins. As a result, the recognition site or the binding properties of target proteins and/or the activity of modified enzymes are altered [9–12]. Importantly, 518 protein kinases have been identified in the human kinome [13]. They are pivotal regulators of cell signalling cascades and networks and represent important putative targets for the development of inhibitors with potential therapeutic application [14–17]. Based on large-scale proteomics analysis performed on the HeLa cell line, a peak of protein phosphorylation appears at the onset of mitosis whereas a drastic reduction of phosphopeptides is observed in late mitosis (anaphase, telophase) [18–20]. Such modulation in protein regulation profoundly alters the behaviour of a significant proportion of proteins. Cdk1-cyclin B is one of the most active and critical complexes during mitosis. It orchestrates the G_2_/M transition and phosphorylates a plethora of M-phase regulators. In anaphase, cyclin B is released from the complex and degraded by the anaphase-promoting complex/cyclosome (APC/c), leading to inactivation of Cdk1 and mitosis exit [21]. It is admitted that phosphorylation of proteins by Cdks coordinates cell division as well as essential cellular processes such as transcription [22], mRNA splicing [23, 24] and translation [25–28]. However, it is not yet understood if Cdks regulate post-transcriptional mechanisms involved in coordinating the expression of mRNAs coding for cell cycle regulators.

STAU2 is a double-stranded RNA-binding protein that is expressed as 4 protein isoforms (52, 56, 59 and 62 kDa) generated by differential splicing of the *STAU2* gene [29, 30]. In mammals, the *STAU2* gene is highly expressed in brain and heart [29] and ubiquitously expressed in all tested cell lines. STAU2 is a component of ribonucleoprotein complexes [29, 31, 32] involved in microtubule-dependent mRNA transport in many species [29, 30, 33–41]. Interestingly, chemical induction of long term depression in hippocampal neurons causes a reduction in the amount of Stau2 in dendrites allowing the release of Stau2-bound mRNAs and their translation on polysomes [40]. Therefore, STAU2 can sequester sub-populations of mRNAs and allow their release and local translation according to cell needs. In addition to transport, STAU2 was shown to increase the translation of reporter proteins [42] or decay of mRNA [43]. In a high throughput experiment, STAU2 was also found to be required for differential splicing [44]. Using a genome-wide approach, we found that STAU2-bound mRNAs code for proteins involved in catabolic process, post-translational protein modifications, RNA metabolism, splicing, intracellular transport, and translation [45, 46]. Accordingly, STAU2 was linked to multiple cell processes. Stau2 down-regulation in neurons impairs mRNA transport, causes dendritic spines defects and prevents hippocampal long term depression [30, 34, 40]. In addition, Stau2 induces neural stem cell differentiation [47, 48]. Similarly, stau2 is required for survival and migration of primordial germ cells [37] in zebrafish, while it is involved in anterior endodermal organ formation in *Xenopus* [49]. In chicken, STAU2 down-regulation reduced cell proliferation with no evidence of cell death or apoptosis [50]. We recently showed that STAU2 down-regulation increases DNA damage in human cells and promotes apoptosis when cells are challenged with DNA-damaging agents [51]. However, not much is known about STAU2 regulation, although phosphorylation may account for the control of at least some of its functions. Indeed, in Xenopus oocytes, stau2 was shown to be transiently phosphorylated by the mapk pathway during meiotic maturation, a time period that coincides with the release of anchored RNAs from their localization at the vegetal cortex [33]. In rat neurons, the activity-stimulated transport of Stau2-containing complexes in dendrites of neurons is dependent on Mapk activity [35]. Stau2 contains a docking site for Erk1/2 in the RNA-binding domain inter-region and this site is required for proper transport of Stau2-containing complexes [36].

Here, we report that STAU2 is hyperphosphorylated during mitosis and that CDK1 participates in the process. Several phosphorylated amino acids residues were localized as clusters in the C-terminal region of STAU2. Taking together, our results highlight for the first time the fact that the RNA-binding protein STAU2 is finely regulated in a cell-stage-dependant manner.

## Methods

### Plasmids and cloning strategies

The human STAU2^59^ coding sequence was generated by PCR amplification of a commercial clone (ATCC) using sense (ATAAGATATCGCCACCATGCTTCAAATAAATCAGATGTTC) and antisense (ATAAGATATCTTATCAGCGGCCGCCGACGGCCGAGTTTGATTTC) oligonucleotides. The PCR product was then cloned in the retroviral pMSCV puromycin vector after EcoRV digestion and blunt ligation. Subsequently, a C-terminal FLAG_3_ tag was inserted at the Not1 site using complementary sense (5’TCGAGATGGGCGGCCGCGACTACAAAGACCATGACGGTGATTATAAAGATCATGACATCGACTACAAGGATGACGATGACAAGTGATAAGCGGCCGCG3’) and antisense (5’ATTTCGCGGCCGCTTATCACTTGTCATCGTCATCCTTGTAGTCGATGTCATGATCTTTATAATCACCGTCATGGTCTTTGTAGTCGCGGCCGCCCATC3’) oligonucleotides. The same strategy was used to generate STAU2^52^-FLAG_3:_ PCR-amplification from STAU2^59^ with sense (5’TTAAGATATCTCAAGCGGCCGCCTACCTGAAAGCCTTGAATCCTTGC3’) and anti-sense (5’TTAAGATATCTCAAGCGGCCGCCTACCTGAAAGCCTTGAATCCTTGC3’) oligonucleotides, cloning into the pMSCV vector and addition of FLAG_3_ tag at the NotI site. Similarly, STAU2^N-ter^-FLAG_3_ was generated from STAU2^52^ with sense (5’AATTGATATCATGCTTCAAATAAATCAGATGTTCTCAGTGCAG3’) and antisense (5’TTAAGATATCTCATGCGGCCGCCATTAGTGGATGCTTTATAACCAAGTTG3’) oligonucleotides. STAU2^52C-ter^-YFP and STAU2^59C-ter^-mCherry were PCR amplified from STAU2^52^-YFP and STAU2^59^-mCherry, respectively, using sense (5’AATTGATATCATGTTACAACTTGGTTATAAAGCATCCACTAAT3’) and antisense (5’AATTGATATCAGCGGCCGCTTATCACTTGTACAGCTCGTCCATGCCG3’). oligonucleotides.

To construct the P(7) phospho-mutants, DNA fragments of 280 bp containing the 7 mutated residues (T^373^, T^376^, S^384^, S^394^, S^408^, S^423^ and S^426^) were in vitro synthesized (Life Technologies) and cloned in the STAU2^52^- and STAU2^59^-FLAG pMSCV puromycin vector using AanI and MunI restriction enzymes. Threonine and Serine residues were simultaneously substituted for aspartic acid or alanine amino acids to generate the P(7) phospho-mimetic or phospho-null mutants, respectively.

PCR-mediated site specific mutagenesis was used to convert S454, T456, S460 into S454A, T456A, S460A and S454D, T456D, S460D, respectively. The resulting PCR fragments were digested with MunI and DraIII and cloned into pMSCV-STAU2^52^-FLAG_3_ and pMSCV-STAU2^59^-FLAG_3_ to create P(3)- and P(3) + mutants, and into P(7)- and P(7) + vectors to generate P(10)- and P(10) + mutants.

### Cell culture and synchronization

Human cell lines (American Type Culture Collection) were cultured in Dulbecco modified Eagle’s medium supplemented with 10% (*v*/v) fetal bovine serum (Wisent), 100 μg/ml streptomycin and 100 units/ml penicillin (Wisent); here referred to be a complete DMEM. Cells were cultured at 37 °C under a 5% CO_2_ atmosphere. For synchronization in prometaphase, hTert-RPE1 and HeLa cell lines were treated with nocodazole (200 and 60 ng/ml, respectively), for 15 to 18 h. Round shaped mitotic cells were recovered and enriched by gentle shaking of the culture dish (shake-off), then directly collected (hours after release = 0) or re-plated in drug free complete DMEM for kinetics. Cells were synchronized at the G_1_/S border using a double thymidine-block (DTB) protocol [52]. Briefly, hTert-RPE1, HeLa and HCT116 cells were treated with 5, 2,5 and 2 mM thymidine for 16 h and released for 8 h in fresh medium before the second thymidine block was performed for another 16 h. Cells were then washed three times in phosphate buffered saline (PBS) and either collected (hour after treatment = 0) or released in fresh medium for time course. Cells were also synchronized at the G_2_/M phase border using 10 μM of the CDK1 inhibitor, RO-3306 (Enzo Life Sciences) for 18 h or at the metaphase/anaphase transition by paclitaxel (Taxol - Sigma). Cells were then washed three times in PBS and either collected (hours after treatment = 0) or released in fresh medium for different time periods.

### DNA transfection and viral transduction

Closed circular DNA plasmids purification was performed through CsCl-Ethidium bromide gradients protocol to obtain high DNA concentration and maximum purity. For transient expression, cells were transfected with Lipofectamine 2000 according to the manufacturer’s instructions (Invitrogen). Alternatively, plasmids were transfected in ecotropic Phoenix cells, the virus-containing cell supernatant was collected two days after transfection and used to infect cells as previously described [51].

### Colony forming assays

Cells were selected with puromycin as described above. For colony formation assays, 5000 cells in 6-wells plates were allowed to grow for 8 days. Cells were then washed two times with PBS, fixed 10 min directly in the dish using 1% (*v*/v) glutarhaldehyde in PBS, washed two times with PBS and stained with 0,1% (*w*/*v*) crystal violet in PBS for 45 min. After extensive washes in water, plates were dried, scanned and decoloured in a solution containing 10% (*v*/v) acid acetic in distillate water. Colony formation was determined by measuring absorbance at 590 nm. Even if hTert-RPE1 did not form colony, this method was suitable to quantify cell density following control condition based data normalization.

### Sample preparation and gel electrophoresis

Total-cell extracts were prepared and boiled 10 min in lysis buffer (250 mM Tris-Cl pH 6.8, 5% (*w*/*v*) SDS and 40% (*v*/v) Glycerol). Before completing extracts with Laemmli sample buffer, protein concentrations were measured using BCA assays (ThermoScientific) and adjusted equally. Extracts were finally heated at 100 °C for 10 min to denature proteins. For separation, 10 to 20 μg of protein samples were submitted to an 8% or 10% SDS-PAGE prepared from 29:1 acrylamide/bisacrylamide mix. To characterize STAU2, Flag and Rsk-1 gel shit patterns, samples were loaded onto an 8% modified SDS-PAGE prepared from 16:0,215 acrylamide/bisacrylamide mix. Following gel migration, proteins were transferred on nitrocellulose membrane and stained with ponceau red (5 g/L Ponceau S (BioShop) to control transfer and loading.

### Western blot analysis and antibodies

Nitrocellulose membranes were decoloured in distillate water for 10 min, saturated by 5% (*w*/*v*) skim milk in PBS-Tween20 (0,2% (*v*/v)), incubated in primary antibody (see below for antibody preparation) for 2 h or overnight, washed three times for 10 min in PBS-Tween20 (0,2% (*v*/v)), incubated in secondary antibody for 1 h, washed three times for 15 min in PBS-Tween20 (0,2% (*v*/v)), and finally subjected to HRP chemiluminescence reaction. Western blotting data were collected onto X-ray films (Fujifilm).

Primary antibodies were prepared with 1% (*w*/*v*) skim milk in PBS-Tween20 (0,2%). 0,1‰ (*w*/*v*) sodium azide was also added to prevent antibody contamination and allow its long-term conservation. Membranes were incubated 2 h at room temperature with monoclonal anti-cyclin A1 (Sigma) or anti-β-actin (Sigma), or with polyclonal anti-RSK1 (Santa Cruz) antibody. Overnight incubation was required for monoclonal anti-FLAG (Sigma), anti-cyclin B1 (Santa Cruz) and anti-MPM2 (Abcam) antibodies, as well for anti-STAU2 (Sigma) antibody. Secondary antibodies were prepared extemporaneously with 2,5% (*w*/*v*) skim milk in PBS-Tween20 (0,2%). Membranes were incubated at room temperature for 1 h with polyclonal goat anti-mouse (Dako) or anti-rabbit (Dako) HRP-conjugated secondary antibodies.

Other antibodies targeted nucleolin (Abcam), STAU1 [53], GFP (Millipore), RFP (Medical & Biological Laboratories LTD), histone H3 (Abcam), pS28-histone H3 (Millipore), pS10-histone H3 (Millipore), ubiquitin (Millipore), pCDK1/MAPK-substrates (Cell Signaling), pS22-lamina A/C (Cell Signaling), pY15-CDK1 (ThermoScientific), aurora A (Abcam), pT288/T232/T198-aurora A/B/C (Cell Signaling), aurora B (Cell Signaling), pT102/Y204-MAPK/ERK1/2 (Cell Signaling), MAPK/ERK1/2 (Cell Signaling).

### In vitro dephosphorylation assay and phosphoprotein purification

Asynchronous or mitotic cells were prepared in lysis buffer (50 mM Tris-Cl pH 7.5, 150 mM NaCl, 10 mM MgCl_2_, 1% (*v*/v) triton X-100 and Complete EDTA-free protease inhibitor cocktail (Roche)) and lysates were cleared by 15,000 *g* centrifugation for 15 min at 4 °C. 100 to 200 μg of proteins were then incubated in a 100 μl final volume including either 3 U/μl Calf Intestinal Alkaline Phosphatase (CIP, 37 °C for 3 h, New England BioLabs) with 1× buffer, or 8 U/μl Lambda Phosphatase (λPP, 30 °C for 3 h, New England BioLabs) with 1× buffer and 1 mM MnCl_2_. For control conditions, the same quantity of each phosphatases was inactivated according to the manufacturer instructions, and then added with proteins, 50 mM EDTA, 50 mM NaF and 50 mM Na_3_VO_4_ to the 100 μl final volume sample mix. In vitro assayed extracts were completed with Laemmli sample buffer and heated at 100 °C for 10 min to denature proteins before western blotting analysis.

Phosphorylated proteins were also purified by affinity chromatography. For this purpose, lysates from asynchronous or mitotic extracts were submitted to a phosphoprotein purification kit (Qiagen) following the manufacturer instructions. Total cell extracts, flow through and eluates were analysed by western blotting to control and characterize phosphoprotein enrichment.

### Flow cytometry analysis

Cell cycle distribution was determined by Fluorescence Activated Cell Sorting (FACS). Fixed overnight in 70% (*v*/v) ethanol at −20 °C, cells were washed in PBS and resuspended in PBS containing 40 μg/ml propidium iodide and 100 μg/ml RNase A (Sigma). Cells were incubated for 30 min at 37 °C and data was acquired using a BD LSRII apparatus. For each experiment, 10^4^ cells were analyzed.

### Phosphorylation inhibition in vivo

Cells were incubated with nocodazole for 16 h. Cells were then incubated with nocodazole, specific kinase inhibitors and the proteasome inhibitor MG132 (Sigma, 20 uM) for an additional 4 h. Cells were collected and extracts were analyzed by western blotting. Cells were treated with either DMSO (Sigma, 0.1 to 1‰), Purvalanol A (20 uM, Sigma-Aldrich), Roscovitine (100 uM, Millipore), Flavopiridol (2 uM, Cedarlane), RO-3306 (10 uM, Enzo Life Sciences), Alisertib (100 nM, Cedarlane), Barasertib (75 nM, Cedarlane), BI2536 (50 nM, Cedarlane), U0126 (20 uM, Millipore), AZD6244 (40 uM, Symansis), BI-D1870 (20 uM, Cedarlane), SB203580 (20 uM, Sigma-Aldrich), and SP600135 (20 uM, Sigma-Aldrich).

## Results

### Post-translational modification of STAU2 in mitosis

**Fig. 1:**
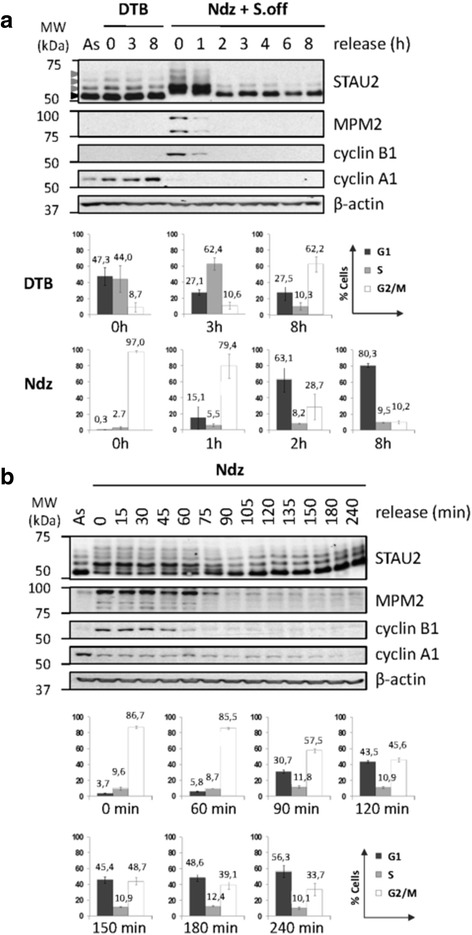
STAU2 is differentially regulated through the cell cycle in hTert-RPE1 cells. **a** hTert-RPE1 cells were synchronized by a double thymidine block (DTB) or a nocodazole arrest (Ndz) followed by shake off (S.off). Cells were then released in a fresh medium for different time periods as indicated (Rel (h)). Asynchronous (As) cells were collected as controls. Protein extracts from synchronized cells were analyzed by SDS-PAGE and western blotting to investigate STAU2 pattern migration and expression of cell cycle markers (MPM2 and cyclins). β-actin was used as loading control. As control of synchronization, the percentage of cell population in the G_1_, S or G_2_/M phases was determined by FACS analysis (*n* = 3)(see also Additional file 1). **b** STAU2 migration dynamics was examined in hTert-RPE1 cells. Cells were blocked in prometaphase with nocodazole (Ndz) without shake-off, released in fresh medium and harvested every 15 min (Rel (min)). Extracts from untreated asynchronous (As) and nocodazole-treated cells were analyzed by western blotting. The percentage of cell population within the G_1_, S or G_2_/M phases was determined by FACS (*n* = 3). In both (**a**) and (**b**), Western blots are representatives of three independently performed experiments that showed similar profiles. Error bars represent the standard deviation

To determine if specific regulation of human STAU2 occurs during cell division, expression of endogenous STAU2 isoforms was monitored through the cell cycle. Untransformed hTert-RPE1 cells were first arrested at the G_1_/S transition by a double thymidine block (DTB) and then released in fresh medium to allow cell progression through the S and G_2_ phases (Fig 1a). In parallel, nocodazole was used to arrest cells in prometaphase (Figs 1a and b). Mitotic cells were recovered by a gentle shake-off, replated and released from the block by fresh medium to reach late mitosis and the G_1_ phase. Cells were then harvested at different time points after release and analyzed by western blotting (Fig 1) and FACS (Additional file 1). G_1_/S-arrested DTB-treated cells successfully reached the S and G_2_-phases about 3 h and 8 h post-release, respectively. Protein analysis indicated that STAU2 expression is stable during these phases of the cell cycle. In contrast, prometaphase-arrested nocodazole-treated cells showed a strong shift in STAU2 isoforms migration that disappeared 2 h post-release (Fig 1a). A time course experiment indicated that the slow migration bands returned to a fast migration pattern 90 min post-release (Fig 1b). This corresponds to cell entry into the G_1_-phase (Additional file 1B). Indeed, at these time points, the mitosis markers MPM2 and cyclin B1 were no longer visible (Fig 1b). Similar results were obtained in the transformed HeLa cell line (Additional files 2, 3): stable STAU2 expression during the S and G_2_ phases of the cell cycle and a slow migration pattern of STAU2 isoforms during mitosis. Immunodetection of cell cycle markers and FACS analyses confirmed the phases of the cell cycle.

**Fig. 2:**
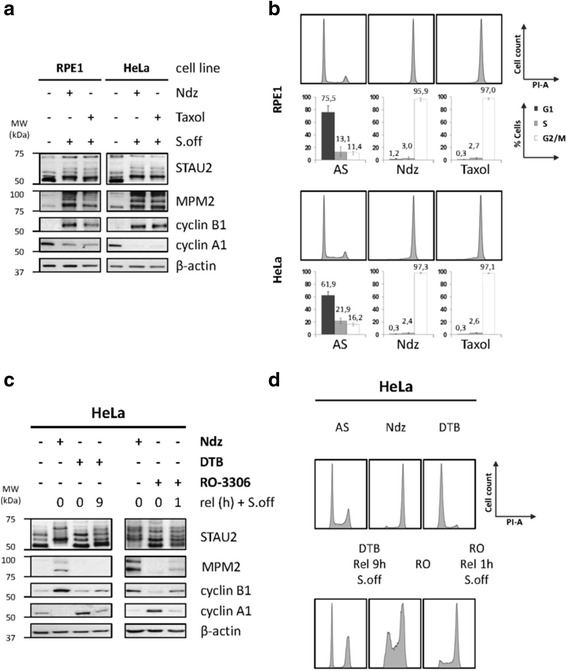
Post-translational modification of STAU2 in mitosis. **a** hTert-RPE1 and HeLa cells were synchronized in mitosis with either nocodazole (Ndz) or paclitaxel (Taxol) and enriched by shake off (S.off). Protein extracts from synchronized cells were analyzed by SDS-PAGE and western blotting to investigate STAU2 migration pattern and expression of cell cycle markers (MPM2 and cyclins). β-actin was used as loading control. **b** As control of synchronization, the percentage of cell population in the G_1_, S or G_2_/M phases was determined by FACS analysis (*n* = 3). **c** HeLa cells were synchronized either in prometaphase by nocodazole (Ndz), in G_1_/S transition by double-thymidine block (DTB) or in late G_2_ by the CDK1 inhibitor RO-3306 (RO-3306) and released from the block for the indicated time periods to reach mitosis. Protein extracts from synchronized cells were analyzed by SDS-PAGE and western blotting to investigate STAU2 migration pattern and expression of mitotic markers (MPM2 and cyclins). β-actin was used as loading control. **d** As control of synchronization, the percentage of cell population in the G_1_, S or G_2_/M phases was determined by FACS analysis (*n* = 3). Error bars represent the standard deviation

To confirm that the slow migration of STAU2 isoforms during mitosis was not a non-specific effect of nocodazole treatment, we synchronized M-phase cells by three additional approaches and compared STAU2 isoform migration to that obtained in the presence of nocodazole. First, paclitaxel (taxol) was used to block hTert-RPE1 and HeLa cells in mitosis, as confirmed by western blotting (Fig 2a) and FACS analysis (Fig 2b). This drug caused a delay in STAU2 migration as did nocodazole (Fig 2a). Then, HeLa cells were arrested at the G_1_/S transition by a DTB and release for 9 h to reach mitosis. In parallel, cells were arrested in late G_2_ by the CDK1 inhibitor [54] RO-3306 and released for 1 h until appearance of sufficient amounts of round shaped mitotic cells. In both cases, mitotic cells were enriched by gentle shake-off and cell extracts were prepared for western blotting and FACS (Figs 2c and d, respectively). Following DTB and RO-3306 releases, cells reached mitosis inconsistently. Mitotic markers were weakly expressed and mitotic synchronization was less efficient, as compared to nocodazole-treated cells. Nevertheless, both treatments induced a comparable slow pattern of migration (Fig 2c). These results confirmed that the slow migration profile of STAU2 is mitosis-specific and suggest that STAU2 is post-translationally modified during mitosis.

### The slow migrating pattern of STAU2 is observed in all tested cell lines

**Fig. 3:**
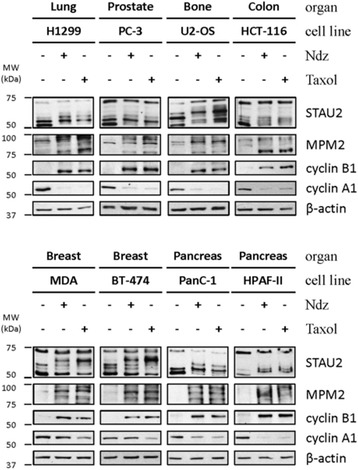
Mitosis-specific migration pattern of STAU2 is observed in all tested cell lines. Eight cell lines derived from different organs were synchronized (+) by either nocodazole (Ndz) or paclitaxel (Taxol) and cell extracts were analyzed by western blotting to investigate STAU2 migration pattern and expression of cell cycle markers (MPM2 and cyclins). β-actin was used as loading control. Synchronization was confirmed by FACS (see Additional file 4). Western blots are representatives of three independently performed experiments that showed similar profiles

Our data indicate that STAU2 is modified during mitosis in both untransformed and transformed cell lines derived from two different tissues, normal retina and tumor from cervix, respectively. To determine how universal is this property, eight additional cancer cell lines derived from different organs were synchronized with nocodazole or paclitaxel (taxol) as above. Cell extracts were analyzed by western blotting (Fig 3) and FACS (Additional file 4). Interestingly, the slow migrating bands of STAU2 isoforms were observed in all tested cell lines with both drugs indicating that STAU2 post-translational modification is ubiquitous and independent of cell origin. Efficient mitotic synchronization was confirmed in all cell lines by cell cycle markers MPM2 and cyclin B1 and by FACS.

### STAU2 is hyper-phosphorylated in M-phase

**Fig. 4:**
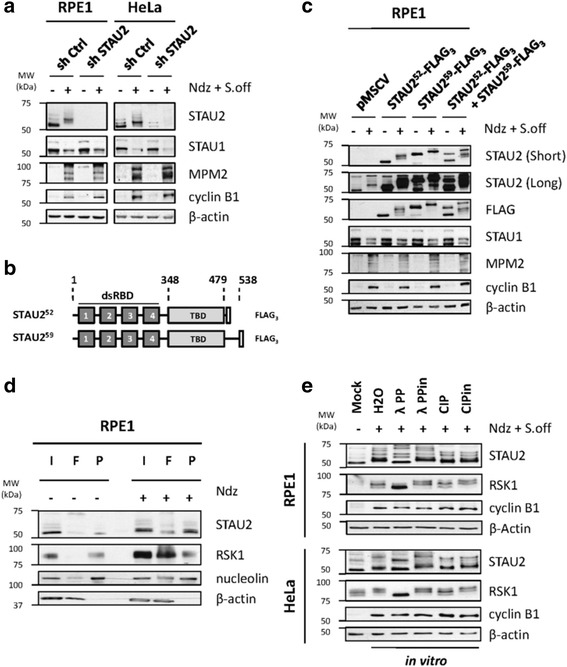
STAU2 is hyperphosphorylated during the M-phase. **a** shRNA control (shCtrl) or against STAU2 (shSTAU2) were cloned in a retrovirus vector to infect hTert-RPE1 and HeLa cells. Cells were synchronized in prometaphase with nocodazole and mitotic cells were enriched by gentle shake off (Ndz + S.off). Protein extracts from asynchronous (−) and synchronized cells (+) were analyzed by western blotting to detect STAU2 migration and cell cycle markers (MPM2 and cyclins). β-actin was used as loading control. **b** Schematic representation of STAU2 expression vectors, STAU2^52^-FLAG_3_ and STAU2^59^-FLAG_3_. Dark gray boxes, double-stranded RNA-binding domains (dsRBD); light gray boxes, tubulin-binding domain (TBD); white boxes, FLAG_3_. **c** hTert-RPE1 cells infected with viruses expressing the empty pMSCV vector (pMSCV), STAU2^52^-FLAG_3_, STAU2^59^-FLAG_3_ or both were synchronized (+) by nocodazole and shake off (Ndz + S.off). Migration of STAU2 proteins was detected by SDS-PAGE and western blotting. Both endogenous and overexpressed STAU2^59^-FLAG_3_ were analyzed with anti-STAU2 antibody, while anti-FLAG antibody was used to specifically recognize FLAG_3_-tagged STAU2 isoforms. Mitotic marker accumulation was assessed with anti-MPM2 and anti-cyclin B1 antibodies. Loading was normalized with β-actin antibody. **d** Asynchronous (−) and nocodazole-treated (Ndz)(+) hTert-RPE1 cells were lysed and protein extracts were subjected to separation on phospho-columns. Input from total extracts (I), flow through (F) and phospho-eluates (P) were analyzed by western blotting using anti-STAU2. Anti-RSK1, anti-nucleolin and anti-β-actin antibodies were used as controls for phosphorylated and unphosphorylated proteins, respectively. **e** Protein extracts from nocodazole-treated (+) hTert-RPE1 and HeLa were incubated in vitro with either water (H2O), Lambda Phosphatase (λPP), inactivated Lambda Phosphatase (λPPin), Calf Intestinal Alkaline Phosphatase (CIP) or inactivated Calf Intestinal Alkaline Phosphatase (CIPin). STAU2 phosphorylation status was analyzed by SDS-PAGE and western blotting. Untreated cells (Mock) were used as control for dephosphorylated STAU2. All western blots are representatives of three independently performed experiments that showed similar profiles

Using RNA interference, we first proved that the bands observed during interphase and mitosis corresponded to STAU2 isoforms (Fig 4a). HeLa and hTert-RPE1 cells were infected with control shRNA or shRNA against STAU2, incubated in nocodazole-complemented medium and shaken-off to collect prometaphase arrested cells. Asynchronous cells were used as controls. Cell extracts were analyzed by SDS-PAGE (Fig 4a). In both cell lines, bands recognized by the STAU2 antibody disappeared as a consequence of infection with shRNA against STAU2, authenticating the shifted bands as STAU2 proteins. STAU1 was used as control to confirm the specificity of the RNA interference. Its partial degradation in mitosis is consistent with our previous data [55]. Then, human STAU2^52^-FLAG_3_ and STAU2^59^-FLAG_3_ (Fig 4b) were expressed in hTert-RPE1 cells and their migration in extracts of untreated and nocodazole-treated cells was observed by western blotting using anti-STAU2 and anti-FLAG antibodies (Fig 4c). As expected, both overexpressed isoforms appeared as slow migrating bands in M-phase-enriched cell extracts.

Differential migration pattern of proteins is usually caused by protein post-translational modifications such as phosphorylation. Interestingly, Xstaufen was previously shown to be phosphorylated during meiotic maturation in Xenopus oocyte [33]. Therefore, two different approaches were used to determine if STAU2 is phosphorylated during mitosis. First, untreated and nocodazole-treated cell extracts were loaded on phospho-specific columns. Column-trapped phosphoproteins were eluted and analyzed by western blotting (Fig 4d). Proteins in the flow-through were also analyzed. A significant fraction of STAU2 was found in the column eluate of nocodazole-treated cells and showed as expected a slow migration pattern. STAU2 fast-migrating bands were found in the flow-through. In untreated cells, a significant amount of fast-migrating bands was found in the column eluate indicating a basal phosphorylation pattern of STAU2 isoforms in interphase. RSK1, nucleolin and β-actin were used as controls for phosphorylated and non-phosphorylated proteins, respectively. Then, hTert-RPE1 and HeLa cell extracts from nocodazole-synchronized cells were treated with λ protein phosphatase or calf intestinal alkaline phosphatase (CIP) in vitro prior to analysis by SDS-PAGE (Fig 4e). While STAU2 isoforms in the mitotic cell extracts showed the slow migration pattern, treatment with λ phosphatase completely abolished the migration shift and treatment with CIP had a partial effect (Fig 4e). Dephosphorylation of STAU2 isoforms was specific since heat-inactivation of phosphatases prior to incubation restored the slow migration of the bands. The phosphorylation pattern of RSK1 was used as control.

### STAU2 is phosphorylated in the C-terminal region

**Fig. 5:**
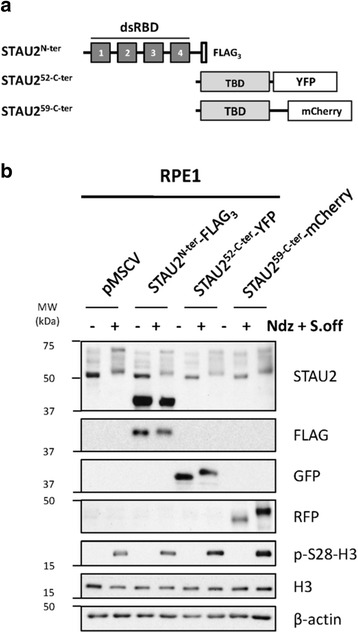
STAU2 is phosphorylated in the tubulin-binding domain during mitosis. **a** Schematic representation of STAU2 deletion mutants. Dark gray boxes, double-stranded RNA-binding domains (dsRBD); light gray boxes, tubulin-binding domain (TBD); white boxes, yellow fluorescent protein (YFP) or red fluorescent protein (mCherry). **b** hTert-RPE1 cells were infected with retrovirus expressing either STAU2^N-ter^-FLAG_3_, STAU2^52-C-ter^-YFP or STAU2^59-C-ter^-mCherry and protein extracts were analyzed by western blotting to detect STAU2 phosphorylation pattern migration (anti-STAU2, anti-FLAG, anti-GFP and anti-RFP) and expression of mitotic markers (p-S28-H3). β-actin was used as loading control. Western blots are representatives of three independently performed experiments that showed similar profiles

To localize the sites of phosphorylation, we generated three truncated mutants: human STAU2^N-ter^-FLAG_3_ coding for the four N-terminal dsRBDs, human STAU2^52-C-ter^-YFP coding for the STAU2^52^ tubulin-binding domain (TBD) and short C-terminal region and human STAU2^59-C-ter^-mCherry coding for the STAU2^59^ TBD and long C-terminal region (Fig 5a). hTert-RPE1 cells were transfected with plasmids coding for the deletion mutants, synchronized or not in mitosis with nocodazole and analyzed by western blotting (Fig 5b). While the endogenous STAU2 proteins showed a typical gel shift pattern in mitosis, no difference in the migration of STAU2^N-ter^-FLAG_3_ was observed between asynchronous and mitotic cell extracts. In contrast, slow migrating bands appeared in mitotic cell extracts following expression of STAU2^52-C-ter^-YFP and STAU2^59-C-ter^-mCherry. These results indicate that a region in the C-terminal half of the protein that is common to both STAU2^52^ and STAU2^59^ contains phosphorylation site(s) responsible for their slow migration pattern in mitosis.

### Multiple residues are phosphorylated in the STAU2 C-terminal region during M-phase

**Fig. 6:**
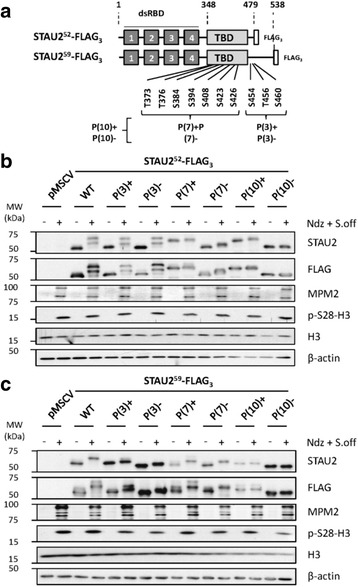
Multiple residues are phosphorylated in the TBD during mitosis. **a** Schematic representation of STAU2 showing the 4 double-stranded mRNA-binding domains (dsRBD) and the tubulin-binding domain (TBD). Large-scale mass spectrometry experiments previously identified ten amino acid residues that are specifically phosphorylated during the M-phase. Three, seven and ten residues were simultaneously mutated to generate phospho-mimetic (P(3)+, P(7)+, P(10)+) and phospho-null (P(3)-, P(7)-, P(10)-) STAU2^52^-FLAG_3_ and STAU2^59^-FLAG_3_ mutants in the retroviral pMSCV vector. **b**,**c** hTert-RPE1 were infected with viruses expressing empty pMSCV, wild type (WT) or phospho-STAU2^52^-FLAG_3_ (**b**) or phospho-STAU2^59^-FLAG_3_ (**c**) mutants and selected with puromycin. Asynchronous (−) and mitotic (+)(Ndz + S.off) cell extracts were analyzed by western blotting to detect STAU2 migration and mitotic markers (MPM2 and p-S28-H3). All western blots are representatives of three independently performed experiments that showed similar profiles

Large-scale analysis of phospho-proteomes identified several mitosis-specific STAU2 phospho-residues [56]. Interestingly, seven of them were located within TBD and three in the short C-terminal region (Fig 6a). We thus muted as clusters the seven and the three amino acid residues found in TBD and C-terminal domain, respectively, as well as all ten residues. Residues were substituted for either aspartic acid or alanine to respectively generate phospho-mimetic (P(7)+; P(3)+; P(10)+) or phospho-null (P(7)-; P(3)-; P(10)-) STAU2^52^-FLAG_3_ and STAU2^59^-FLAG_3_ mutants (Fig 6a).

hTert-RPE1 cells were infected with viruses expressing either wild type (WT) STAU2^52^-FLAG_3_, the phospho-mimetic or the phospho-null mutant. Their migration patterns were analyzed by western blotting in extracts from both mitotic and unsynchronized cells (Fig 6b). STAU2^52^-FLAG_3_ was found as two slow migrating bands when extracted from mitotic cells as compared to unsynchronized cells, indicating the presence of two differentially phosphorylated populations of STAU2 in mitosis. Essentially the same migration pattern was observed with the WT, P(3) + and P(3)- mutants suggesting that residues in the P3 cluster did not contribute much to STAU2 phosphorylation pattern in mitosis. In contrast, a single slow migrating band was observed with the phospho-mimetic P(7) + mutants in both asynchronous and mitotic cells, suggesting that at least some residues are phosphorylated in this cluster. Interestingly, whereas the phospho-null P(7)- mutant exhibited the fast migrating pattern in asynchronous cells, as expected, it however showed a slight shift in mitosis, suggesting that other amino acids are also phosphorylated in mitosis. Since a similar shift was not detected with the P(10)- mutant, it is suggests that site(s) in the P3 cluster may accounted for the difference. Altogether, these results mapped the major mitotic phosphorylation residues within the P7 cluster and suggested that residues in the P3 cluster may also contribute to STAU2 phosphorylation. A slightly different pattern of phosphorylation was observed for STAU2^59^-FLAG_3_ (Fig 6c). First, STAU2^59^-FLAG_3_ showed a single phosphorylated band in mitosis. Second, both the P(3) and P(7) mutants showed altered migration patterns in asynchronous and mitotic cells as compared to the wild type protein, and both phospho-null P(3)- and P(7)- proteins slightly shifted in mitotic cells as compared to asynchronous cells. Nevertheless, as observed above for STAU2^52^-FLAG_3_ P(10) mutants, STAU2^59^-FLAG_3_ P(10) mutants did not shift during mitosis indicating that the major phosphorylation sites are located within the P3 and P7 clusters. These results suggest a more important role for residues in the P3 cluster for STAU2^59^-FLAG_3_ than for STAU2^52^-FLAG_3_.

### STAU2 phosphorylation depends on CDK1

**Fig. 7:**
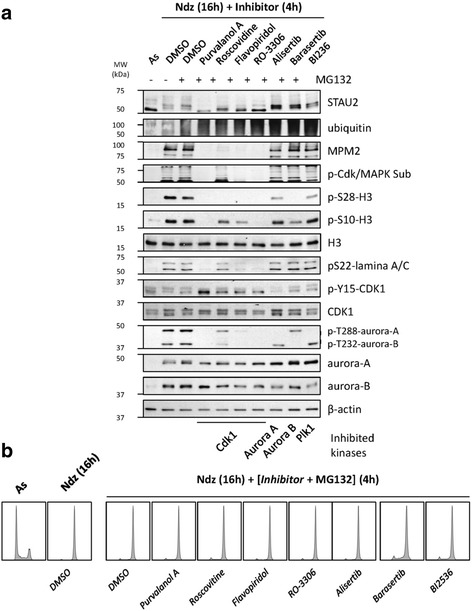
STAU2 is phosphorylated by Cdk1 during mitosis in hTert-RPE1 cells. **a** hTert-RPE1 cells were incubated with nocodazole (Ndz) for 16 h in the absence (DMSO) or presence of specific kinase inhibitors for 4 h as indicated. Asynchronous (As) cells were used as controls. Cells were treated with the proteasome inhibitor MG132 (+) to keep cells in mitosis. STAU2 migration on SDS-gels was analyzed by western blotting. Cell markers were used to confirm specific inhibition of kinases. **b** Cell synchronization was confirmed by FACS. Western blots and FACS are representatives of three independently performed experiments that showed similar profiles

To further explore the STAU2 phosphorylation pathway in mitosis, hTert-RPE1 cells were synchronized in prometaphase with nocodazole and exposed for 4 h to kinase inhibitors in the presence of the proteasome inhibitor MG132 to prevent cyclin B degradation and subsequent mitotic exit. Several drugs were tested that specifically targeted CDK1 (Purvalanol A, Roscovitine, Flavopiridol, RO-3306), aurora-A (Alisertib), aurora-B (Barasertib), and polo-like kinase 1 (PLK1)(BI2536). Cell extracts were analyzed by western blotting (Fig 7a) and FACS (Fig 7b). Inhibition of CDK1 by any of the four kinase inhibitors prevented the phosphorylation-mediated shift migration of STAU2 in mitosis (Fig 7a). As controls, we showed that CDK1 inhibition prevented the phosphorylation of histone H3, lamina A/C and aurora kinases, known targets of CDK1 (Fig 7a). As further controls, CDK1 inhibition did not allow accumulation of MPM2 or phospho-CDK/MAPK substrates. Importantly, upon these conditions, cells were still in the mitotic phase as monitored by FACS (Fig 7b). In contrast, none of the other kinase inhibitors changed the phosphorylation status of STAU2. As evidence of their inhibitory effect and specificity, aurora-A and PLK-1 inhibitors abolished aurora-A phosphorylation on threonine288, and aurora-B inhibitors seriously diminished aurora-B threonine232 phosphorylation (Fig 7a).

Similar experiments were reproduced in HeLa cells (Additional file 5). As shown above, CDK1 inhibitors prevented STAU2 phosphorylation whereas inhibition of aurora A, aurora B or PLK1 had no effect. Additional inhibitors that targeted MAP2K-MEK1/2 (U0126, AZD6244), RSK-1 (BI-D1870), p38MPK (SB203580) and SAPK/JNK (SP600135) were tested in both hTert-RPE1 (Additional file 6A) and HeLa (Additional file 6B) cells. None of these inhibitors affected STAU2 phosphorylation pattern. Inhibition of the MAPKs was monitored with anti-phospho-ERK1/2 (threonine202 and tyrosine204) and through RSK-1 migration pattern.

## Discussion

Cell division is a complex process that required a plethora of overlapping mechanisms to precisely control the timing of each step [1]. Post-translational modification of regulatory proteins is a well-documented process that insures spatial and temporal activation and/or inhibition of protein activity [3, 11]. Among them, protein kinases and phosphatases are at the heart of mechanisms that are essential for progression through mitosis. In this manuscript, we add an additional player to the repertoire of proteins specifically phosphorylated during mitosis. We showed that STAU2 is phosphorylated by a mitotic kinase at the beginning of mitosis and is dephosphorylated as cell exit mitosis. This phosphorylation is ubiquitous, occurs on serine and threonine residues within the tubulin-binding and C-terminal domains of STAU2 and is CDK1-dependant. Unfortunately, the consequence of STAU2 phosphorylation in mitosis is still unclear. However, we excluded the possibility that STAU2 phosphorylation controls cell proliferation, STAU2 protein half-life, interaction with known RNA and protein partners and subcellular localization (see below). One possibility is that, in unstressed cells, STAU2 phosphorylation plays a subtle role that escapes detection but becomes crucial in special conditions (e.g. asymmetric cell division, cell stresses). Alternatively, the putative mechanism that relies on STAU2 phosphorylation might be a backup strategy for the fine tuning of a specific function during cell division. For example, in drosophila, neuroblast asymmetric cell division depends on the differential localization of prospero (pros) mRNA and protein in one of the two progenies. While pros mRNA localization is mediated by Staufen, targeting of Pros protein is not. In Staufen mutant, pros mRNA is no longer localized but still cell fate defects have not been detected in the nervous system, suggesting that Staufen-mediated pros mRNA localization is redundant to Pros protein segregation [57].

### Post-translational modifications of STAU2 during mitosis

Our results indicate that STAU2 is specifically phosphorylated during mitosis in human cells. This is reminiscent of the temporal phosphorylation of Xstau proteins during meiotic maturation in Xenopus [33]. It was suggested that phosphorylation of Xstau may alter its RNA-binding activity to allow the release and/or expression of Xstau-bound RNAs. In oocytes, Xstau proteins are transiently phosphorylated by the MAPK pathway. Our pharmacological studies in human cells do not support the possibility that the MAPK pathway phosphorylates STAU2 during mitosis. They rather indicate that cyclin-dependent kinase 1 is involved in this process. We do not exclude the possibility that members of the MAPK pathway may also phosphorylate STAU2 during mitosis but that the process is likely masked by the CDK1-mediated hyper-phosphorylation. Alternatively, phosphorylation of STAU2 by MAPK may occur in other phases of the cell cycle. Indeed, large-scale phosphoproteome analyses and phospho-columns separation (Fig 4d) indicate that STAU2 is also phosphorylated during interphase. Interestingly, STAU2 was shown to harbor a docking site for ERK1/2 [36]. Its presence in the RNA-binding domain inter-region of the protein would be consistent with a role in modulating its RNA binding activity.

CDK1 is the major kinase regulating mitosis. In human, CDK1-cyclin B phosphorylates more than 70 substrates required for mitosis progression [4]. Our results add a member of the RNA-binding proteins family to the list of CDK1 substrates. Previous phosphoproteome analyses identified 10 amino acid residues that are more likely to be phosphorylated in mitoses than during interphase [56]. Our data indicated that phosphorylation of these residues is responsible for the migration shift of STAU2 isoforms during mitosis. Interestingly, our data are consistent with a differential regulation of the P(3) and P(7) clusters and different phosphorylation mechanisms for the two STAU2 isoforms. It is possible that the two isoforms are differently localized in the cells during mitosis or that they are part of alternative complexes that changes their accessibility for CDK1. However, the exact pattern in the phosphorylated populations and the dynamic and timing of phosphorylation in each complex are still unknown. Many CDK1-mediated phosphorylated residues show the S/T-P-x-K/R consensus sequence [11]. Although none of the phosphorylated residues in the P3 and P7 clusters of STAU2 have a perfect match with the consensus sequence, 4 residues in the P7 clusters and all three residues in the P3 cluster have the S/T-P consensus sequence. It is possible that the 3 other sites contain atypical non-S/T-P consensus motifs as previously described in proteins such as vimentin, desmin and myosin II [58]. We do not exclude the alternative possibilities that other kinases also phosphorylated STAU2 in mitosis but do not significantly contribute to the migration shifts observed in mitosis.

### Functions of phosphorylated STAU2 in mitosis

The consequence of STAU2 phosphorylation in mitosis is unclear. Using the phospho-mimetic and phospho-null mutants, we first excluded a role in the RNA-binding capacity of STAU2 (data not shown). This was expected since the phosphorylated residues were mapped within the C-terminal region of the protein, a region that lacks RNA-binding activity. In addition, we showed that STAU2 interaction with ribosomes or the RNA-decay factor UPF1 was not altered by phosphorylation (data not shown), suggesting that its functions in the post-transcriptional regulation of gene expression are not affected. It was previously shown that the human paralog STAU1 interacts with these factors via its TBD [59, 60]. Obviously, STAU2 phosphorylation in TBD does not regulate these processes.

We then excluded a role for phosphorylation in STAU2 stability (Additional file 7). We had noticed a decrease in the amounts of STAU2 in the G_1_ phase of the cell cycle as compared to those in G_2_, suggesting that STAU2 is partly degraded in mitosis (Fig. 1; Additional file 2A). Phosphorylation could have been used as a signal for protein stability, but this is not the case. STAU2 partial degradation in mitosis likely involved the proteasome since its amount was increased in the presence of the proteasome inhibitor MG132 (Additional file 7). This is reminiscent of STAU1 which is partly degraded during its transit through mitosis as a consequence of its binding to the E3-ubiquitin ligase APC/C and its degradation by the proteasome [55].

We further excluded the possibility that phosphorylation in mitosis changes STAU2 sub-cellular localization (data not shown). We previously showed that STAU2 migrates in dendrites on the microtubule network [29]. Similar results were obtained with STAU1 [61] and the molecular determinant for tubulin-binding was mapped within TBD [60]. Therefore, STAU2 phosphorylation in TBD might prevent STAU2-microtubule association. Indeed, STAU2 was not found on microtubules during mitosis in hamster BHK and mouse MEF cells [62], although it partly colocalizes with spindle at MI and MII during mouse oocyte meiosis. However, the STAU2 phospho-null mutant was not better in associating with the mitotic spindle than the WT STAU2 or its phospho-mimetic mutant (data not shown).

Finally, it was previously shown that STAU2 may be involved in the control of cell division. Indeed, STAU2 depletion leads to decreased cell proliferation and small eye phenotypes in chicken embryos [50], and increases the number of post-mitotic neurons in rats [47, 48]. Similarly, overexpression of the paralog STAU1 impairs cell proliferation by affecting mitosis entry [55]. Thus, mitosis-specific phosphorylation of STAU2 might be advantageous to regulate some aspects of cell division. However, overexpression of STAU2 phospho-mimetic and phospho-null mutants in the untransformed hTert-RPE1 cells led to the same proliferation pattern as that of the cells that overexpressed the WT protein (Additional file 8).

## Conclusions

Altogether, our results indicate that STAU2 is hyper-phosphorylated in mitosis suggesting that it may be a novel actor in mitosis regulation and that post-transcriptional regulation of gene expression may be linked to cell cycle pathways in proliferative cells.

## Additional files

Additional file 1: Synchronization of hTert-RPE1 cells – Controls for experiments shown in Fig. 1. (A) hTert-RPE1 cells were synchronized by a double thymidine block (DTB) or a nocodazole arrest followed by shake off (Ndz + S.off). Cells were then released in fresh medium for different time periods as indicated. Asynchronous (As) cells were collected as controls. **(B)** Cells were blocked in prometaphase with nocodazole and shake off (Ndz + S.off), released in fresh medium and harvested every 30 min. Cell synchronization was analyzed by FACS. These results are representatives of three independently performed experiments that showed similar profiles. (PDF 132 kb)

Additional file 2: STAU2 is differentially regulated through the cell cycle in HeLa cells. HeLa cells were synchronized by a double thymidine block (DTB) or a nocodazole arrest (Ndz) followed by shake off (S.off). Cells were then released in a fresh medium for different time periods as indicated (Rel (h)). Asynchronous (As) cells were collected as controls. **(A)** Protein extracts from synchronized cells were analyzed by SDS-PAGE and western blotting to investigate STAU2 phosphorylation pattern migration and expression of mitotic markers (MPM2 and cyclins). β-actin was used as loading control. **(B)** As control of synchronization, the percentage of cell population in the G_1_, S or G_2_/M phases was determined by FACS analysis. Error bars represent the standard deviation. *n* = 3. (PDF 237 kb)

Additional file 3: STAU2 dephosphorylation dynamics was examined in HeLa cells. HeLa cells were blocked in prometaphase with nocodazole (Ndz), released in fresh medium and harvested every 15 min (Rel (min)). **(A)** Extracts from untreated asynchronous (As) and nocodazole-treated cells were analyzed by western blotting. **(B)** The percentage of cell population within the G_1_, S or G_2_/M phases was determined by FACS. Error bars represent the standard deviation. *n* = 3. (PDF 248 kb)

Additional file 4: STAU2 is phosphorylated in mitosis in all tested cell lines – controls for experiments shown in Fig. 3 **.** Eight cell lines derived from different organs were synchronized (+) by either nocodazole (Ndz) or paclitaxel (Taxol). Cells were collected after a gentle shake off and analyzed by FACS. *n* = 3. (PDF 140 kb)

Additional file 5: STAU2 is phosphorylated by CDK1 during mitosis in HeLa cells. HeLa cells were incubated with nocodazole (Ndz) for 16 h in the absence (DMSO) or presence of specific kinase inhibitors for 4 h as indicated. Asynchronous (As) cells were used as controls. Cells were treated with the proteasome inhibitor MG132 (+) to keep cells in mitosis. STAU2 migration on SDS-gels was analyzed by western blotting. Cell markers were used to confirm specific inhibition of kinases. Western blots are representatives of three independently performed experiments that showed similar profiles. (PDF 422 kb)

Additional file 6: MAPK does not phosphorylated STAU2 in mitosis. hTert-RPE1 **(A)** and HeLa **(B)** cells were incubated with nocodazole (Ndz) for 16 h in the absence (DMSO) or presence of specific kinase inhibitors for 4 h as indicated. Asynchronous (As) cells were used as controls. Cells were treated with the proteasome inhibitor MG132 (+) to keep cells in mitosis. STAU2 migration on SDS-gels was analyzed by western blotting. Cell markers were used to confirm specific inhibition of kinases. Western blots are representatives of three independently performed experiments that showed similar profiles. (PDF 416 kb)

Additional file 7: STAU2 phosphorylation does not modulate protein degradation. hTert-RPE1 cells were infected with retroviruses expressing either empty pMSCV, STAU2^52^-FLAG_3_ wild type (WT), phospho-mimetic STAU2^52^-FLAG_3_ (P(10)+) or phospho-null STAU2^52^-FLAG_3_ (P(10)-). Cells were synchronized in prometaphase with nocodazole and released from the block for 6 h (Ndz + Rel). During release, prometaphase cells were also treated (+) or not (−) with cyclohexamide (CHX) to prevent protein synthesis and with (+) or without (−) the proteasome inhibitor MG132 to prevent protein degradation. Cell extracts were collected and amounts of STAU2 analyzed by western blotting. β-actin was used as loading control. Western blots are representatives of three independently performed experiments that showed similar profiles. (PDF 165 kb)

Additional file 8: STAU2 phosphorylation does not regulate cell proliferation. hTert-RPE1 cells were infected with retroviruses expressing either empty pMSCV (pMSCV), STAU2^52^-FLAG_3_ (WT), phospho-mimetic (+) or phospho-null (−) mutants. **(A)** Same amounts of cells were plated and allowed to growth for 5 days in a colony assay. **(B)** Cell proliferation was quantified by crystal violet staining. *n* = 3. **(C)** Western blot analysis indicated that the amounts of each STAU2 overexpressed-protein are similar and slightly above that of the endogenous protein. (PDF 335 kb)

## Abbreviations

APC/c: Anaphase-promoting complex/cyclosome
CDK: Cyclin-dependent kinase
CIP: Calf-intestinal phosphatase
DMSO: Dimethyl sulfoxide
dsRBD: Double-stranded RNA-binding domain
DTB: Double-thymidine block
ERK: Extracellular signal-regulated kinase
FACS: Fluorescence-activated cell sorting
GFP: Green-fluorescent protein
HRP: Horseradish peroxidase
MAPK: Mitogen-activated protein kinase
MEK: Mitogen-activated kinase kinase
PBS: Phosphate buffered saline
PLK1: Polo-like kinase 1
Pros: Prospero
RFP: Red-fluorescent protein
RSK: Ribosomal S6 kinase
SAPK/JNK: Stress-activated protein kinase/c-jun kinase
SDS-PAGE: Sodium dodecyl sulfate-polyacrylamide gel electrophoresis
STAU2: Staufen2
TBD: Tubulin-binding domain
YFP: Yellow fluorescent protein
λPP: Lambda protein phosphatase
