# Correction to: The M-phase specific hyperphosphorylation of Staufen2 involved the cyclin-dependent kinase CDK1

**DOI:** 10.1186/s12860-018-0171-2

**Published:** 2018-09-10

**Authors:** Rémy Beaujois, Elizabeth Ottoni, Xin Zhang, Christina Gagnon, Sami Hassine, Stéphanie Mollet, Wildriss Viranaicken, Luc DesGroseillers

**Affiliations:** 1 Present address: UMR PIMIT, Processus Infectieux en Milieu Insulaire Tropical, Université de la Réunion, 97490 Sainte Clotilde, La Réunion France; 2 Département de biochimie et médecine moléculaire, Faculté de médecine, Université de Montréal, 2900 Edouard Montpetit, Montréal, QC H3T 1J4 Canada

## Correction

Following publication of the original article [1], the authors reported a change to one of the author names.

Originally, the fifth author was published as Sami HSine. After publication, the spelling of his name changed to Sami Hassine.
