# Supplementary figures and images for: The M-phase specific hyperphosphorylation of Staufen2 involved the cyclin-dependent kinase CDK1
Source: BMC Cell Biol. 2017 Jul 14;18:25. doi: 10.1186/s12860-017-0142-z (PMC5513041; doi:10.1186/s12860-017-0142-z)

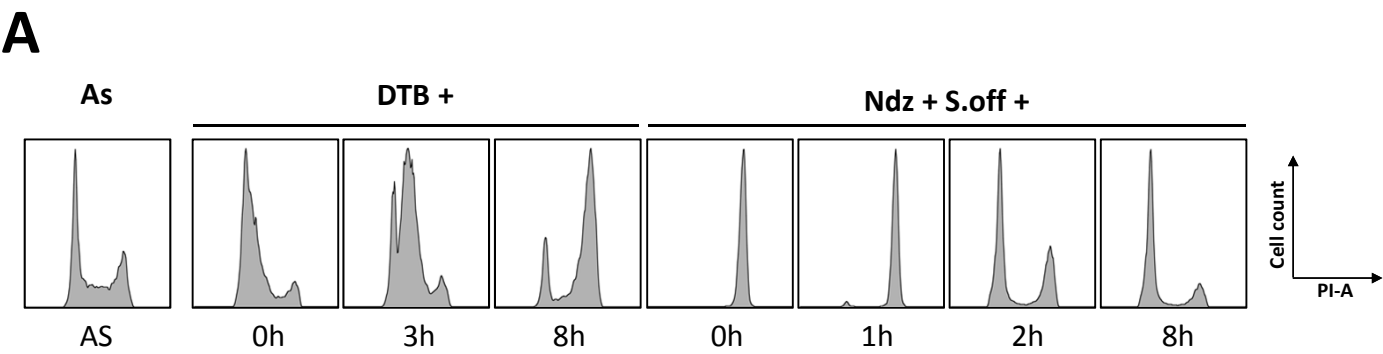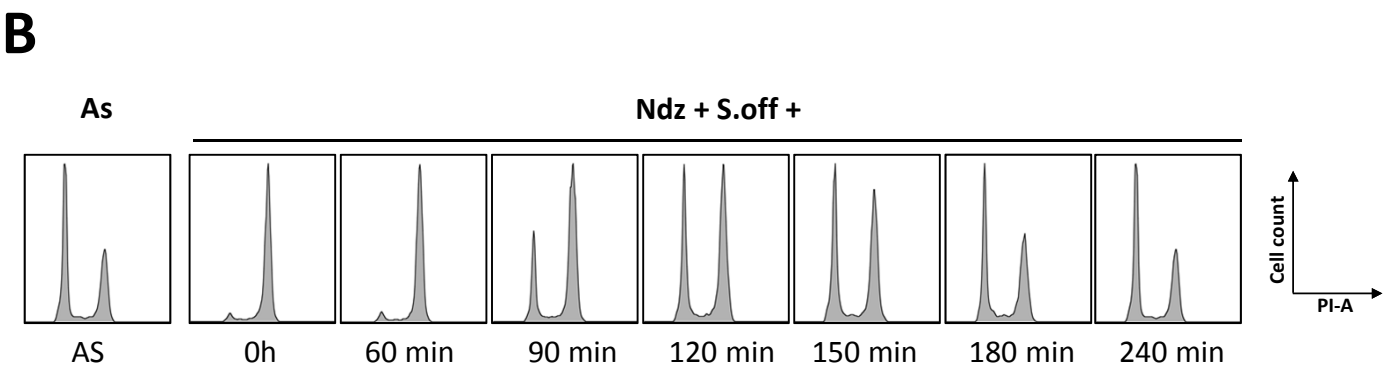

Supplement: Supplementary file 1 — Synchronization of hTert-RPE1 cells – Controls for experiments shown in Fig. 1. (A) hTert-RPE1 cells were synchronized by a double thymidine block (DTB) or a nocodazole arrest followed by shake off (Ndz + S.off). Cells were then released in fresh medium for different time periods as indicated. Asynchronous (As) cells were collected as controls. (B) Cells were blocked in prometaphase with nocodazole and shake off (Ndz + S.off), released in fresh medium and harvested every 30 min. Cell synchronization was analyzed by FACS. These results are representatives of three independently performed experiments that showed similar profiles. (PDF 132 kb) [file 12860_2017_142_MOESM1_ESM.pdf]

**A**

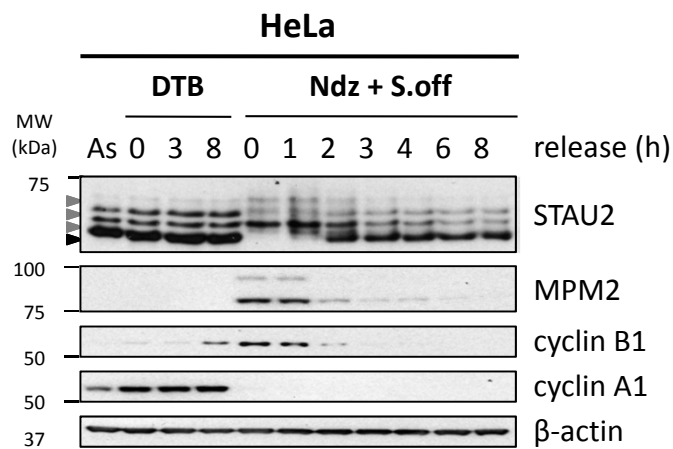

**B**

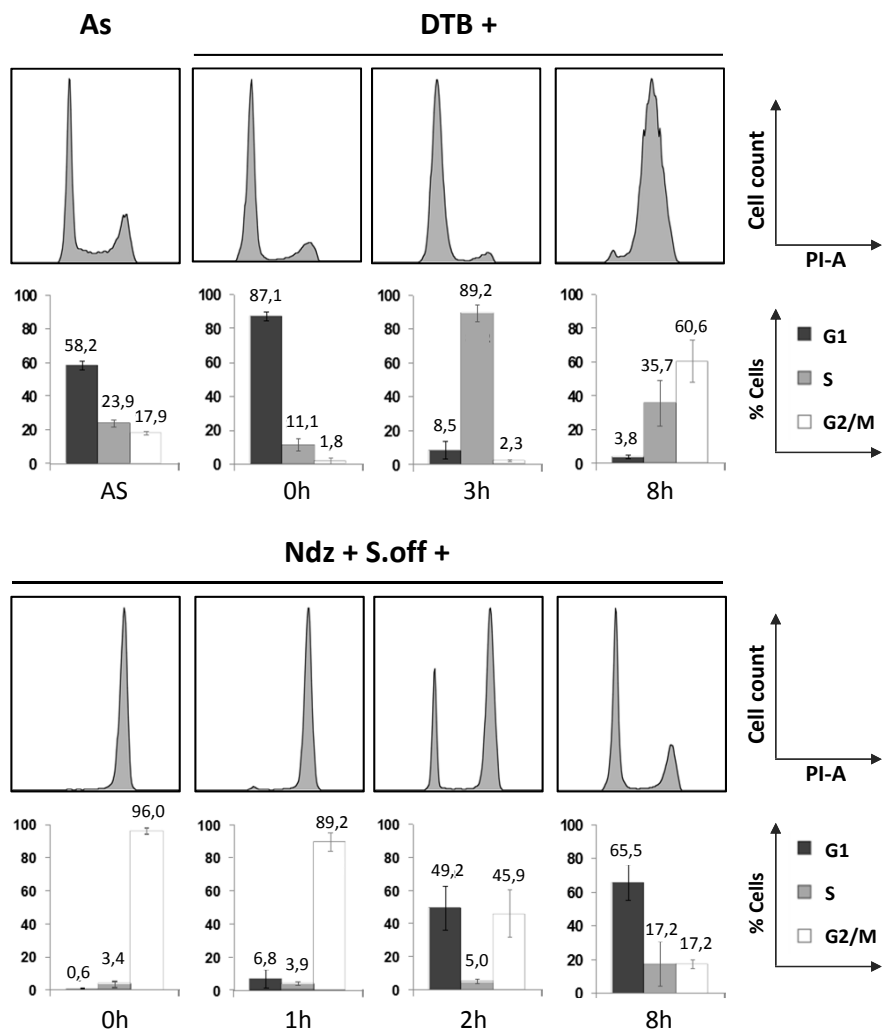

Supplement: Supplementary file 2 — STAU2 is differentially regulated through the cell cycle in HeLa cells. HeLa cells were synchronized by a double thymidine block (DTB) or a nocodazole arrest (Ndz) followed by shake off (S.off). Cells were then released in a fresh medium for different time periods as indicated (Rel (h)). Asynchronous (As) cells were collected as controls. (A) Protein extracts from synchronized cells were analyzed by SDS-PAGE and western blotting to investigate STAU2 phosphorylation pattern migration and expression of mitotic markers (MPM2 and cyclins). β-actin was used as loading control. (B) As control of synchronization, the percentage of cell population in the G1, S or G2/M phases was determined by FACS analysis. Error bars represent the standard deviation. n = 3. (PDF 237 kb) [file 12860_2017_142_MOESM2_ESM.pdf]

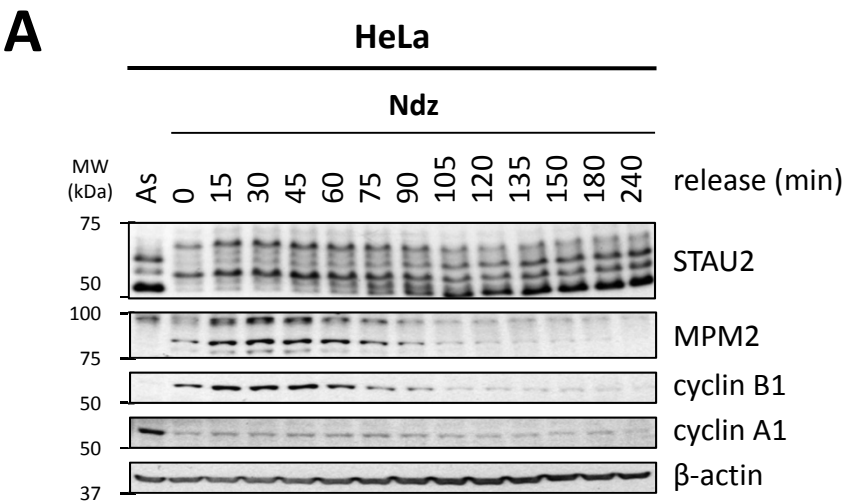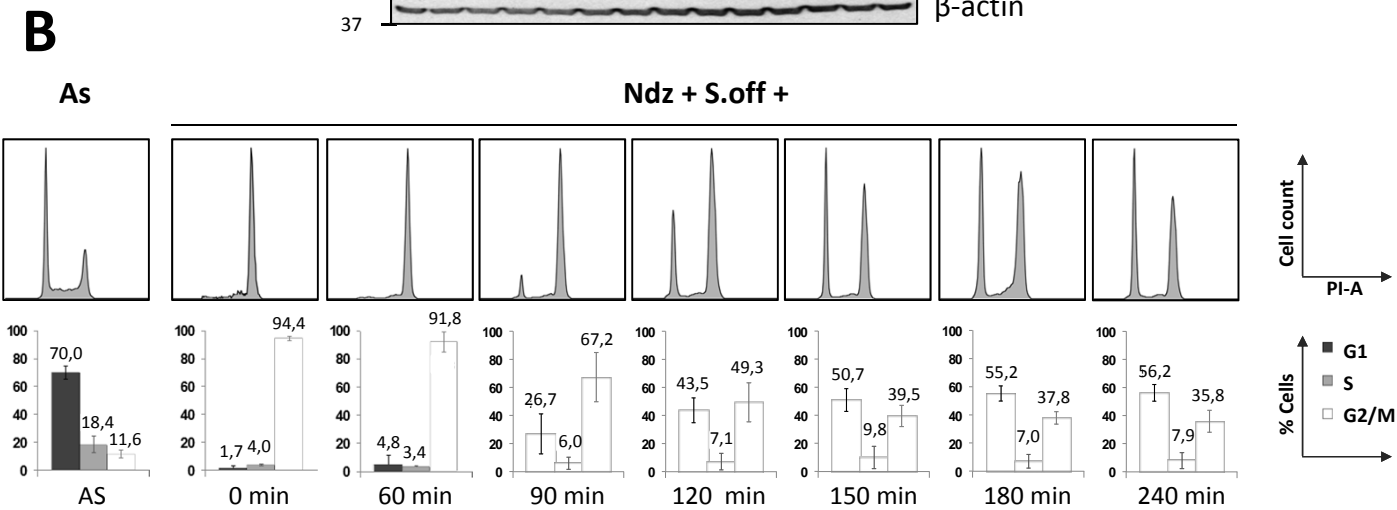

Supplement: Supplementary file 3 — STAU2 dephosphorylation dynamics was examined in HeLa cells. HeLa cells were blocked in prometaphase with nocodazole (Ndz), released in fresh medium and harvested every 15 min (Rel (min)). (A) Extracts from untreated asynchronous (As) and nocodazole-treated cells were analyzed by western blotting. (B) The percentage of cell population within the G1, S or G2/M phases was determined by FACS. Error bars represent the standard deviation. n = 3. (PDF 248 kb) [file 12860_2017_142_MOESM3_ESM.pdf]

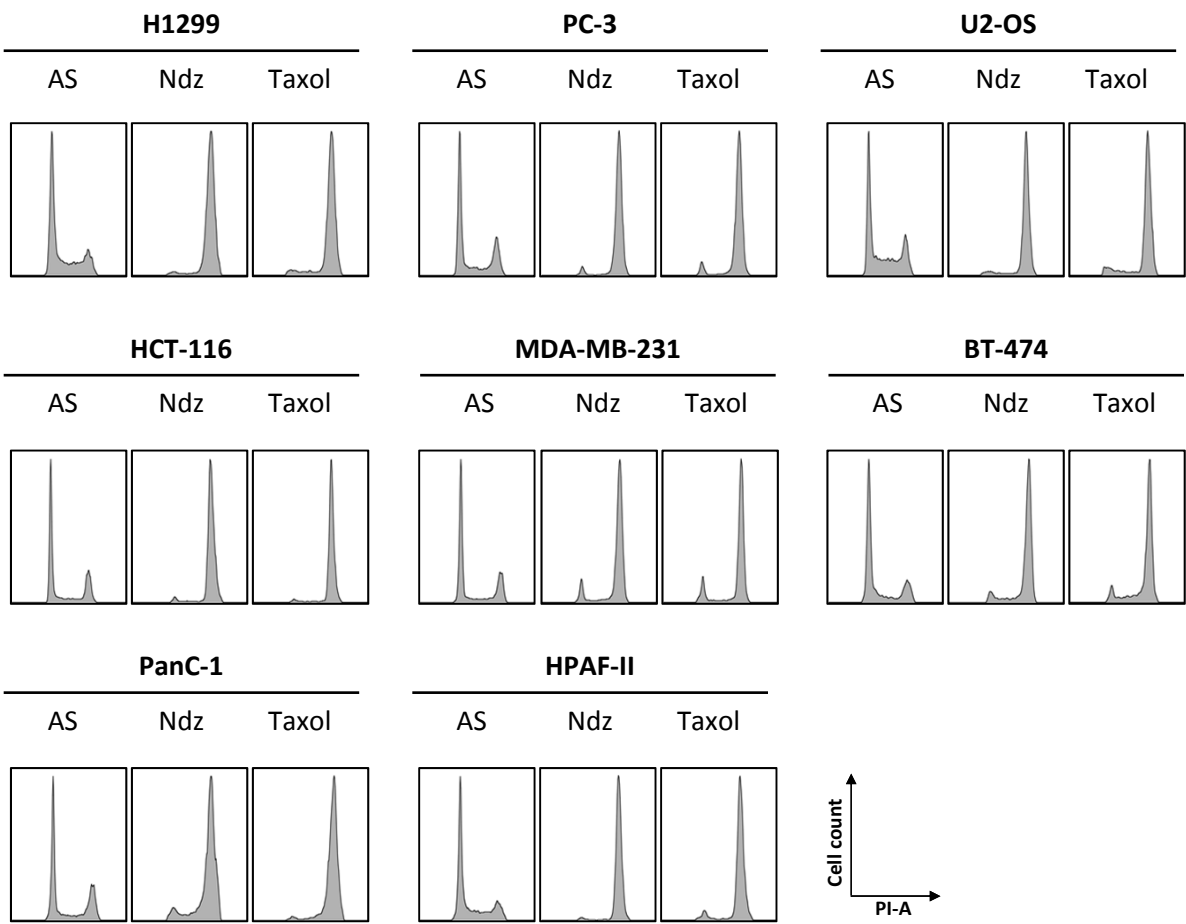

Supplement: Supplementary file 4 — STAU2 is phosphorylated in mitosis in all tested cell lines – controls for experiments shown in Fig. 3 . Eight cell lines derived from different organs were synchronized (+) by either nocodazole (Ndz) or paclitaxel (Taxol). Cells were collected after a gentle shake off and analyzed by FACS. n = 3. (PDF 140 kb) [file 12860_2017_142_MOESM4_ESM.pdf]

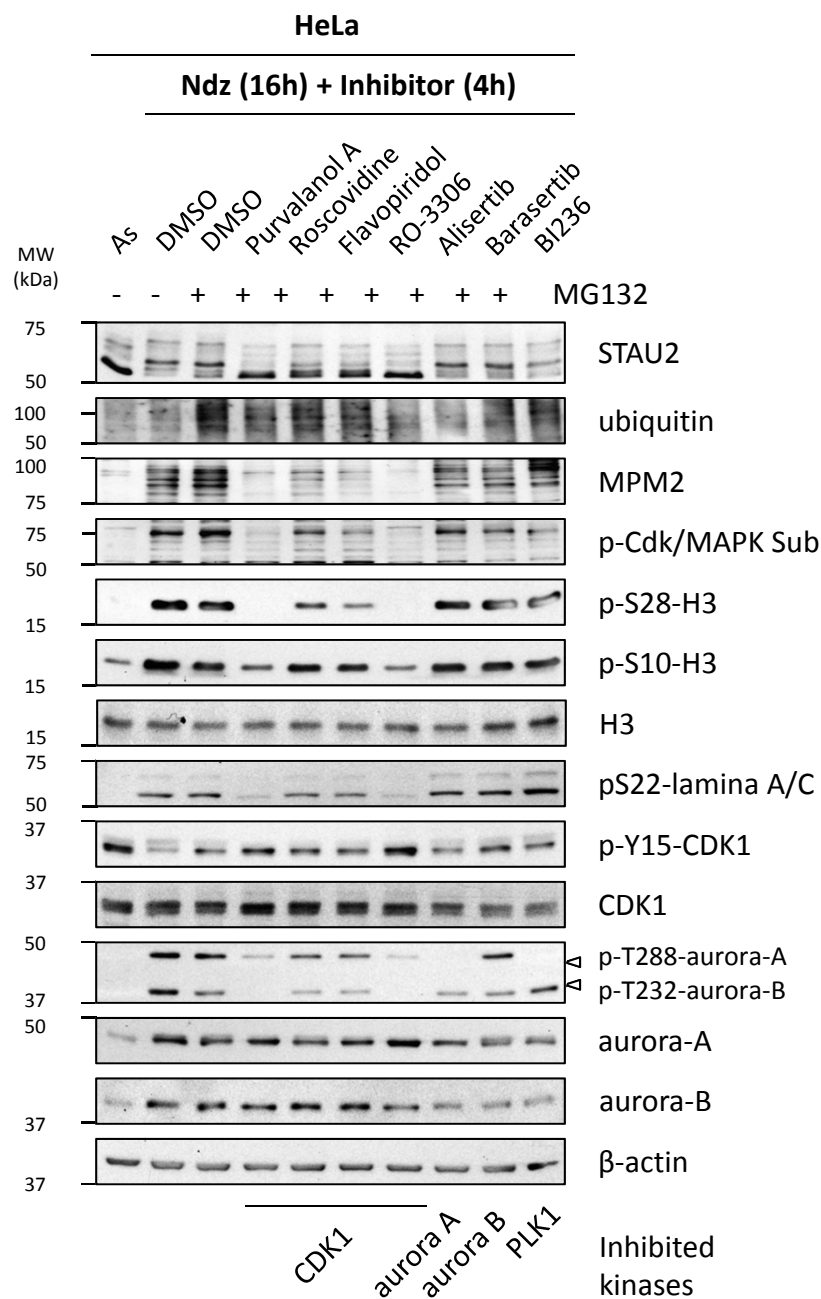

Supplement: Supplementary file 5 — STAU2 is phosphorylated by CDK1 during mitosis in HeLa cells. HeLa cells were incubated with nocodazole (Ndz) for 16 h in the absence (DMSO) or presence of specific kinase inhibitors for 4 h as indicated. Asynchronous (As) cells were used as controls. Cells were treated with the proteasome inhibitor MG132 (+) to keep cells in mitosis. STAU2 migration on SDS-gels was analyzed by western blotting. Cell markers were used to confirm specific inhibition of kinases. Western blots are representatives of three independently performed experiments that showed similar profiles. (PDF 422 kb) [file 12860_2017_142_MOESM5_ESM.pdf]

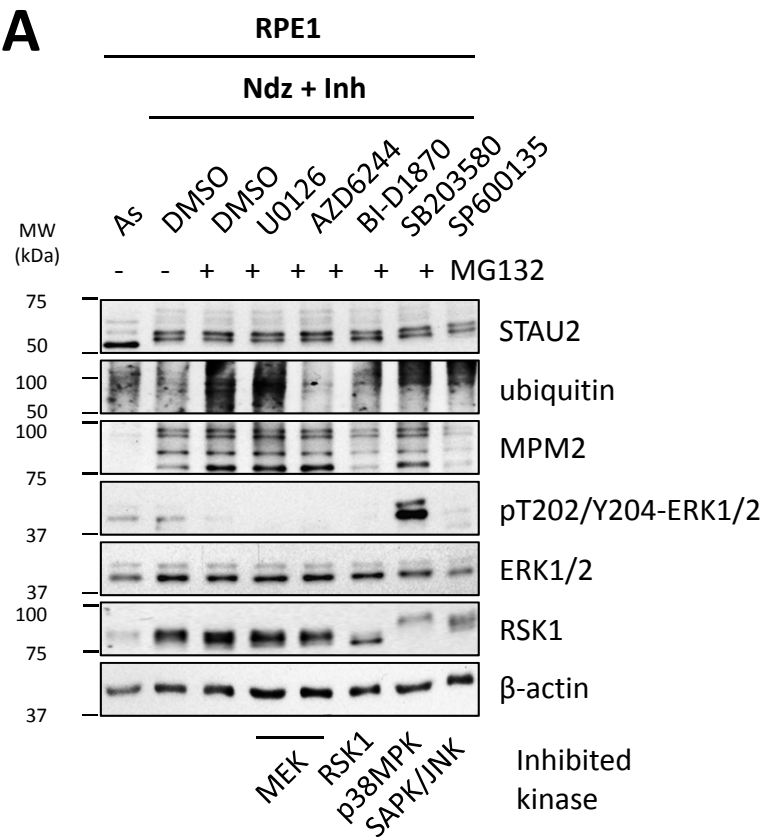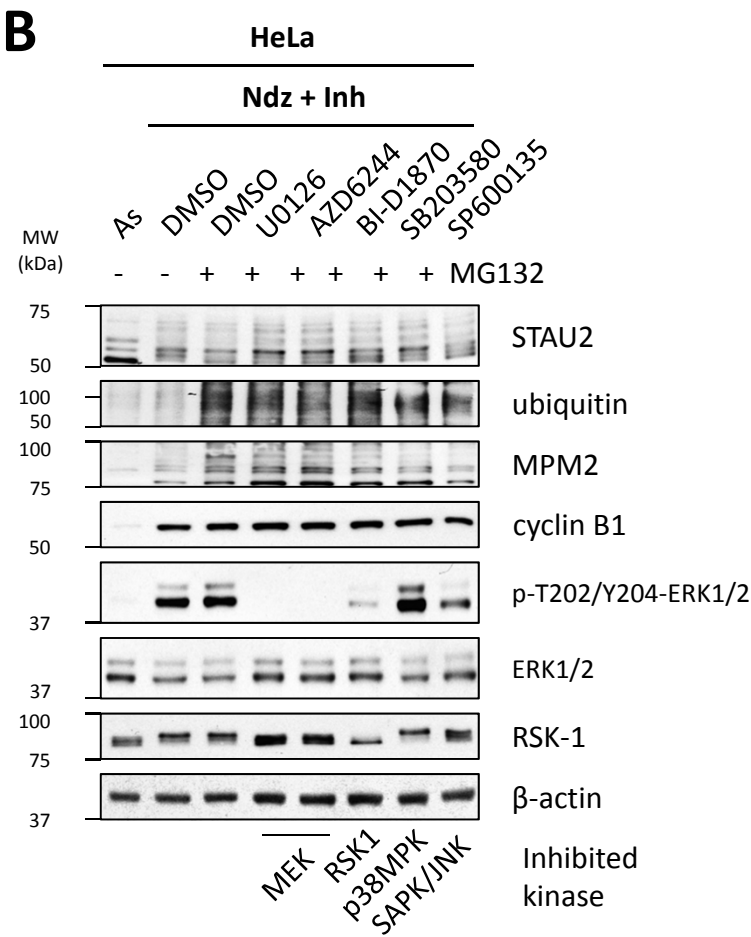

Supplement: Supplementary file 6 — MAPK does not phosphorylated STAU2 in mitosis. hTert-RPE1 (A) and HeLa (B) cells were incubated with nocodazole (Ndz) for 16 h in the absence (DMSO) or presence of specific kinase inhibitors for 4 h as indicated. Asynchronous (As) cells were used as controls. Cells were treated with the proteasome inhibitor MG132 (+) to keep cells in mitosis. STAU2 migration on SDS-gels was analyzed by western blotting. Cell markers were used to confirm specific inhibition of kinases. Western blots are representatives of three independently performed experiments that showed similar profiles. (PDF 416 kb) [file 12860_2017_142_MOESM6_ESM.pdf]

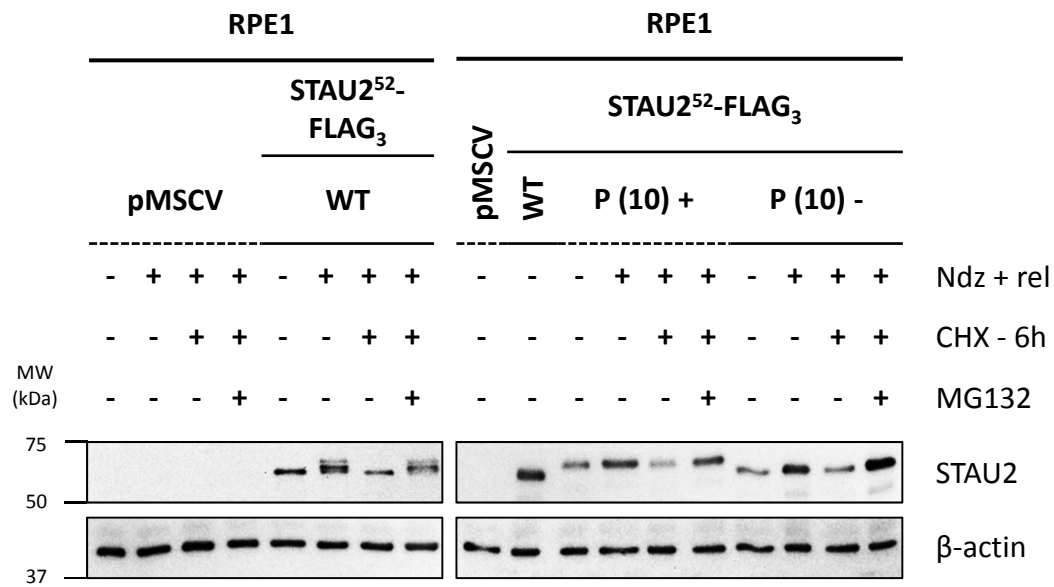

Supplement: Supplementary file 7 — STAU2 phosphorylation does not modulate protein degradation. hTert-RPE1 cells were infected with retroviruses expressing either empty pMSCV, STAU252-FLAG3 wild type (WT), phospho-mimetic STAU252-FLAG3 (P(10)+) or phospho-null STAU252-FLAG3 (P(10)-). Cells were synchronized in prometaphase with nocodazole and released from the block for 6 h (Ndz + Rel). During release, prometaphase cells were also treated (+) or not (−) with cyclohexamide (CHX) to prevent protein synthesis and with (+) or without (−) the proteasome inhibitor MG132 to prevent protein degradation. Cell extracts were collected and amounts of STAU2 analyzed by western blotting. β-actin was used as loading control. Western blots are representatives of three independently performed experiments that showed similar profiles. (PDF 165 kb) [file 12860_2017_142_MOESM7_ESM.pdf]

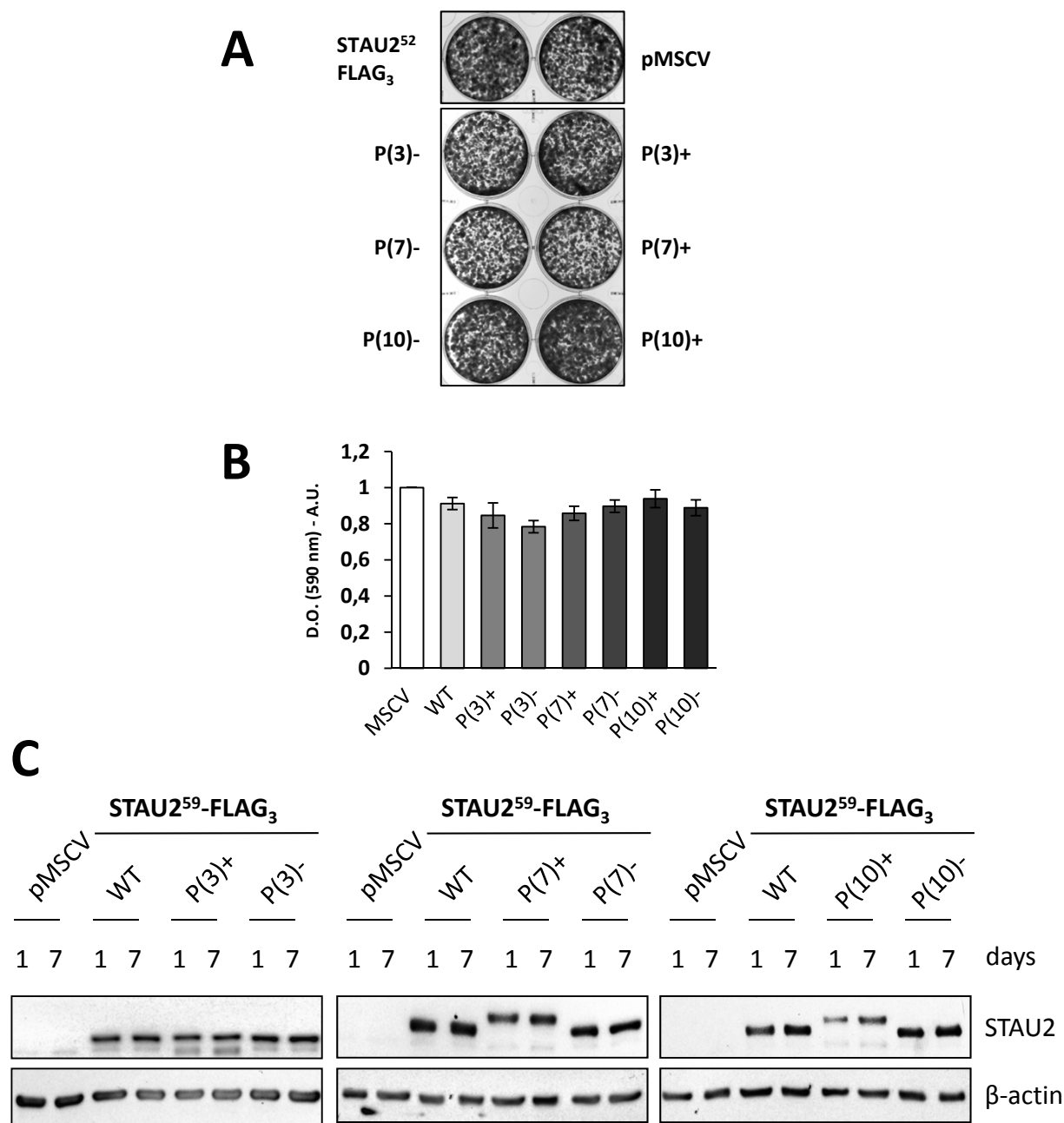

Supplement: Supplementary file 8 — STAU2 phosphorylation does not regulate cell proliferation. hTert-RPE1 cells were infected with retroviruses expressing either empty pMSCV (pMSCV), STAU252-FLAG3 (WT), phospho-mimetic (+) or phospho-null (−) mutants. (A) Same amounts of cells were plated and allowed to growth for 5 days in a colony assay. (B) Cell proliferation was quantified by crystal violet staining. n = 3. (C) Western blot analysis indicated that the amounts of each STAU2 overexpressed-protein are similar and slightly above that of the endogenous protein. (PDF 335 kb) [file 12860_2017_142_MOESM8_ESM.pdf]
